# Total body bone mineral density and various spinal disorders: a Mendelian randomization study

**DOI:** 10.3389/fendo.2023.1285137

**Published:** 2023-10-31

**Authors:** Qingyu Jiang, Haihao Gao, Xudong Shi, Yan Wu, Wentao Ni, Aijia Shang

**Affiliations:** ^1^ Chinese PLA Medical School, Beijing, China; ^2^ Department of Neurosurgery, The First Medical Centre, Chinese PLA General Hospital, Beijing, China; ^3^ Medical School, Nankai University, Tianjin, China; ^4^ Department of Pulmonary and Critical Care Medicine, Peking University People’s Hospital, Beijing, China

**Keywords:** bone mineral density, spine disorders, spinal instability, spinal stenosis, scoliosis spondylolisthesis, spondylolysis, spondylosis

## Abstract

**Introduction:**

Observational studies have yielded inconsistent findings regarding the correlation between bone mineral density (BMD) and various spinal disorders. To explore the relationship between total-body BMD and various spinal disorders further, we conducted a Mendelian randomization analysis to assess this association.

**Methods:**

Two-sample bidirectional Mendelian randomization (MR) analysis was employed to investigate the association between total-body BMD and various spinal disorders. The inverse-variance weighted (IVW) method was used as the primary effect estimate, and additional methods, including weighted median, MR-Egger, simple mode, and weighted mode, were used to assess the reliability of the results. To examine the robustness of the data further, we conducted a sensitivity analysis using alternative bone-density databases, validating the outcome data.

**Results:**

MR revealed a significant positive association between total-body BMD and the prevalence of spondylosis and spinal stenosis. When total-body BMD was considered as the exposure factor, the analysis demonstrated an increased risk of spinal stenosis (IVW odds ratio [OR] 1.23; 95% confidence interval [CI], 1.14–1.32; P < 0.001) and spondylosis (IVW: OR 1.24; 95%CI, 1.16–1.33; P < 0.001). Similarly, when focusing solely on heel BMD as the exposure factor, we found a positive correlation with the development of both spinal stenosis (IVW OR 1.13, 95%CI, 1.05–1.21; P < 0.001) and spondylosis (IVW OR 1.10, 95%CI, 1.03–1.18; P = 0.0048). However, no significant associations were found between total-body BMD and other spinal disorders, including spinal instability, spondylolisthesis/spondylolysis, and scoliosis (P > 0.05).

**Conclusion:**

This study verified an association of total-body BMD with spinal stenosis and with spondylosis. Our results imply that when an increasing trend in BMD is detected during patient examinations and if the patient complains of numbness and pain, the potential occurrence of conditions such as spondylosis or spinal stenosis should be investigated and treated appropriately.

## 1 Introduction

The spine, the central axis of the human body, is a complex and intricate support structure. Its role extends beyond safeguarding the spinal cord and ensuring the execution of various bodily functions (1). With age, spinal columns experience excessive loads and degenerative conditions, such as intervertebral disc herniation, cartilage degeneration in small joints, and ligament calcification, develop. These degenerative changes can lead to spinal stenosis, spondylosis, spinal instability, and vertebral slippage (1–4). This commonly causes symptoms, such as neck and back pain and numbness, which substantially disrupt the quality of daily life (5).

Bone mineralization density (BMD) reflects the mineralization level of bones and is directly correlated with skeletal hardness (6). When bone formation lags behind resorption, significant loss of trabecular bone occurs, predisposing individuals to fractures. This condition is particularly prevalent in postmenopausal women (6, 7). Various methods are available for BMD assessment, with dual-energy X-ray absorptiometry (DXA) being the most widely used. DXA is primarily used to evaluate, diagnose, and treat osteoporosis (8). Total-body BMD measurement by DXA is the most suitable approach for longitudinal assessment of BMD changes in specific skeletal regions, as extensively used in previous research (9). Quantitative ultrasound (QUS) techniques, which are less precise than DXA, are also often used in clinical examinations because of their sensitivity for assessing trabecular bone, which is predominantly present in the calcaneus. QUS does not involve exposure to radiation and is economically viable (10, 11). Only a few studies to date have focused on the relationship between BMD and spinal conditions. Andersen et al. suggest that lower bone density may result in lumbar spondylolisthesis, leading to spinal stenosis (12). On the other hand, Manabe and Fujita have proposed that higher BMD may contribute to articular cartilage damage and ligament ossification, which can then lead to spinal injuries (13, 14). Furthermore, compared to patients with osteoporosis, higher BMD may also be an important risk factor for the development of spinal disorders (15).

Mendelian randomization (MR) is a method for inferring causation that is based on genetic variation. It leverages large-scale genome-wide association study (GWAS) data to explore the causal associations between specific exposures and outcomes (16). Similar to randomized controlled trials, where participants are randomly assigned to experimental or control groups, MR studies involve the “randomization” of one or more alleles associated with risk factors. This approach is used to determine whether carriers of particular genetic variants are more susceptible to development of a specific disease than are noncarriers (17). Unlike traditional observational studies, MR associations remain unaffected by confounding factors. Moreover, GWAS databases are publicly accessible and contain many single nucleotide polymorphisms (SNPs) corresponding to various human traits (18).

We sought to determine the risk factors for common spinal disorders, which is crucial for reducing their incidence and improving prognosis. Given the potential significance of BMD to the pathogenesis of spinal disorders, the present study employed a two-sample MR analysis using genetically linked variations that are strongly correlated with total-body BMD as unconfounded variables for exploring the relationship between total-body BMD and common spinal disorders.

## 2 Materials and methods

### 2.1 Research design

**Figure 1:**
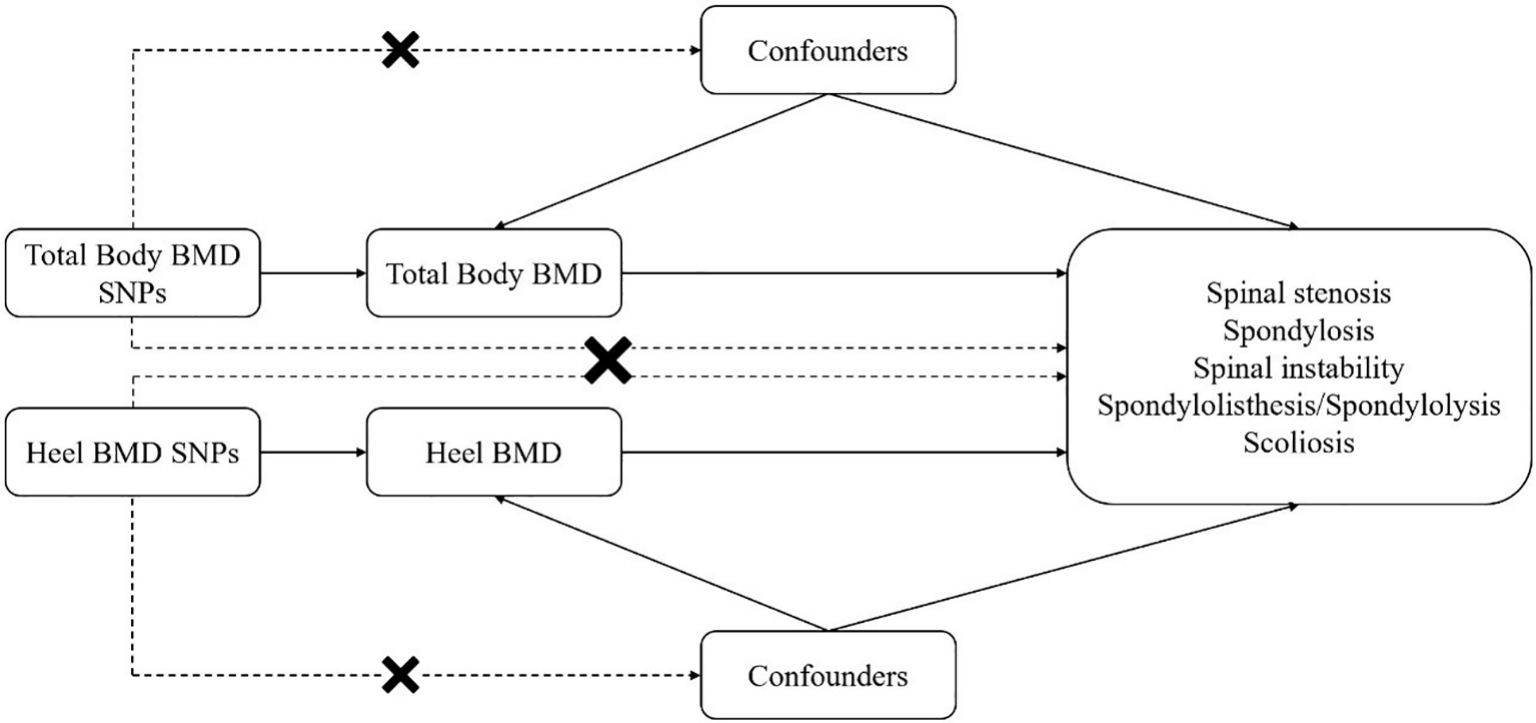
Mendelian randomization (MR) analyses utilizing total-body bone mineral density (BMD) and heel BMD single nucleotide polymorphisms (SNPs) as instrumental variables to establish the causal impact of BMD on different spinal disorders.

The research design is illustrated in **Figure 1**. Initially, we conducted an MR analysis to assess the causal relationship between total-body BMD and various spinal disorders. To validate the reliability of the exposure factor, we used a different set of GWAS data and re-conducted MR analysis to confirm the relationship between heel BMD and spinal disorders. The need to obtain informed consent was waived because the data was deidentified, publicly available database.

### 2.2 Data resource

The GWAS data used in this study included the year of publication, sample size, SNP count, and population information, as detailed in **Table 1**. We made every effort to select the databases with the largest possible sample sizes and the most recent data. The total-body BMD database published in 2018 (9) was derived from European Bioinformatics Institute (EBI) data (ebi-a-GCST005348) integrated into the Integrative Epidemiology Unit (IEU) OPEN GWAS database. This database comprises a sample size of 56,284 individuals of European descent and 13,705,641 SNP markers. For validation purposes, heel BMD data comprises a sample size of 106,254 individuals of European descent and 10,894,596 SNP markers, obtained from the UK Biobank (UKB) data (ukb-a-361) within the IEU GWAS database. And Lumbar spine BMD comprises a sample size of 28,498 individuals and 10,582,897 SNP markers, obtained from the IEU database (ieu-a-982). The outcome data were derived from the FinnGen database.

**Table 1:** Description of GWAS consortia utilized for different phenotypes.

Variable	Sample size	Numbers of SNPs	Population	Diagnostic criteria	Consortium	Year
Total-body BMD	56,284	16,162,733	European	NA	GEFOS	2018
Heel BMD	106,254	10,894,596	European	NA	UKB	2017
Lumbar spine BMD	28,498	10,582,867	Mixed	NA	GEFOS	2015
Spinal stenosis	case: 9,169 control: 164,682	16,380,277	European	ICD10: M48.091	FinnGen	2021
Spondylosis	case: 9,371 control: 164,682	16,380,248	European	ICD10: M47.025+ ICD10: M47.121+ ICD10: M47.225+ ICD10: G54.251	FinnGen	2021
Spinal instability	case: 443 control: 164,682	16,380,219	European	ICD10: M53.295	FinnGen	2021
Spondylolisthesis/Spondylolysis	case: 2,669 control: 164,682	16,380,280	European	ICD10: M43.191 ICD10: Q76.201	FinnGen	2021
Scoliosis	case:1,168 control: 164,682	16,380,270	European	ICD10: M41.992	FinnGen	2021

BMD, bone mineral density; GWAS, genome-wide association study; SNP, single nucleotide polymorphism; UKB, UK Biobank; GEFOS, GEnetic Factors for Osteoporosis.

### 2.3 Instrumental variable selection

Initially, genome-wide SNPs significantly associated with total-body BMD (P < 5 × 10^-8^) were screened from the database. These SNPs were then employed as instrumental variables to assess the causal relationship between exposure and outcomes in MR analysis. SNPs in linkage disequilibrium (LD) were considered independent if their pairwise correlation coefficient (r^2^) was < 0.001 and their distance exceeded 10,000 kb. Confounding factors were screened and removed using PhenoScanner(No confounding factors were found in this study). Instrument strength was estimated using the F-statistic, and F-statistics less than 10 were deemed indicative of weak instrument bias and were subsequently excluded (19, 20).

### 2.4 Statistical analysis

In the MR analysis, the inverse-variance weighted (IVW) method was employed as the primary approach to explore the relationship between total-body BMD and various spinal disorders (21). Heel BMD and Lumbar spine BMD, from different databases, were used as exposure factors, and various methods such as the MR-Egger, weighted median, simple mode, and weighted mode were applied to test the reliability and stability of the results (11, 22). All statistical analyses were conducted using the ‘Two Sample MR’ (version 0.5.6) and ‘Mendelian Randomization’ (version 0.5.1) packages in the R software(version 4.3.1) environment. Statistical significance was set at P < 0.05. significant.

## 3 Results

The positive association between total-body BMD and various spinal disorders was investigated using MR analysis, as presented in **Table 2**. The results obtained after replacement of the exposure data are shown in **Table 3** and **4**.

**Table 2:** Results of MR analysis for total-body BMD on various spinal disorders among GWAS populations.

IVW	SNPs	Beta	Standard error	OR(95%Cl)	P-value
Spinal stenosis	79	0.2069	0.0367	1.2298 (1.1446,1.3214)	P<0.001*
Spondylosis	79	0.2141	0.0347	1.2387 (1.1572,1.3260)	P<0.05*
Spinal instability	79	0.235	0.1284	1.2649 (0.9834,1.6271)	0.0673
Spondylolisthesis/Spondylolysis	79	0.0262	0.0498	1.0265 (0.9311,1.1317)	0.5993
Scoliosis	79	-0.0468	0.0752	0.7423 (0.4545,1.2123)	0.5336

BMD, bone mineral density; IVW, inverse-variance weighting; GWAS, genome-wide association study; MR, Mendelian randomization; SNPs, single nucleotide polymorphisms. (“*” was considered statistically significant).

**Table 3:** Results of MR analysis for heel BMD on various spinal disorders among GWAS populations.

IVW	SNPs	Beta	Standard error	OR(95%Cl)	P-value
Spinal stenosis	126	0.1220	0.0367	1.1296 (1.0514,1.2139)	P<0.001*
Spondylosis	126	0.0955	0.0338	1.1002 (1.0296,1.1756)	P<0.05*
Spinal instability	126	0.0806	0.1157	1.0840 (0.8641,1.3598)	0.4858
Spondylolisthesis/Spondylolysis	126	0.0317	0.0592	1.0322 (0.9190,1.1593)	0.5928
Scoliosis	126	0.0147	0.0687	1.0148 (0.8870,1.1609)	0.831

BMD, bone mineral density; GWAS, genome-wide association study; IVW, inverse-variance weighting; MR, Mendelian randomization; SNPs, single nucleotide polymorphisms. (“*” was considered statistically significant).

**Table 4:** Results of MR analysis for lumbar spine BMD on various spinal disorders among GWAS populations.

IVW	SNPs	Beta	Standard error	OR (95%Cl)	P-value
Spinal stenosis	21	0.3259	0.0464	1.3852 (1.2647,1.5172)	P<0.001*
Spondylosis	21	0.2617	0.0413	1.2992 (1.1981,1.4087)	P<0.001*
Spinal instability	21	-0.0943	0.1862	0.9100 (0.6317,1.3109)	0.6124
Spondylolisthesis/ Spondylolysis	21	0.0491	0.0786	1.0504 (0.9004,1.2254)	0.532
Scoliosis	21	-0.1256	0.1216	0.8820 (0.6950,1.1193)	0.3016

BMD, bone mineral density; GWAS, genome-wide association study; IVW, inverse-variance weighting; MR, Mendelian randomization; SNPs, single nucleotide polymorphisms. (“*” was considered statistically significant).

**Figure 2:**
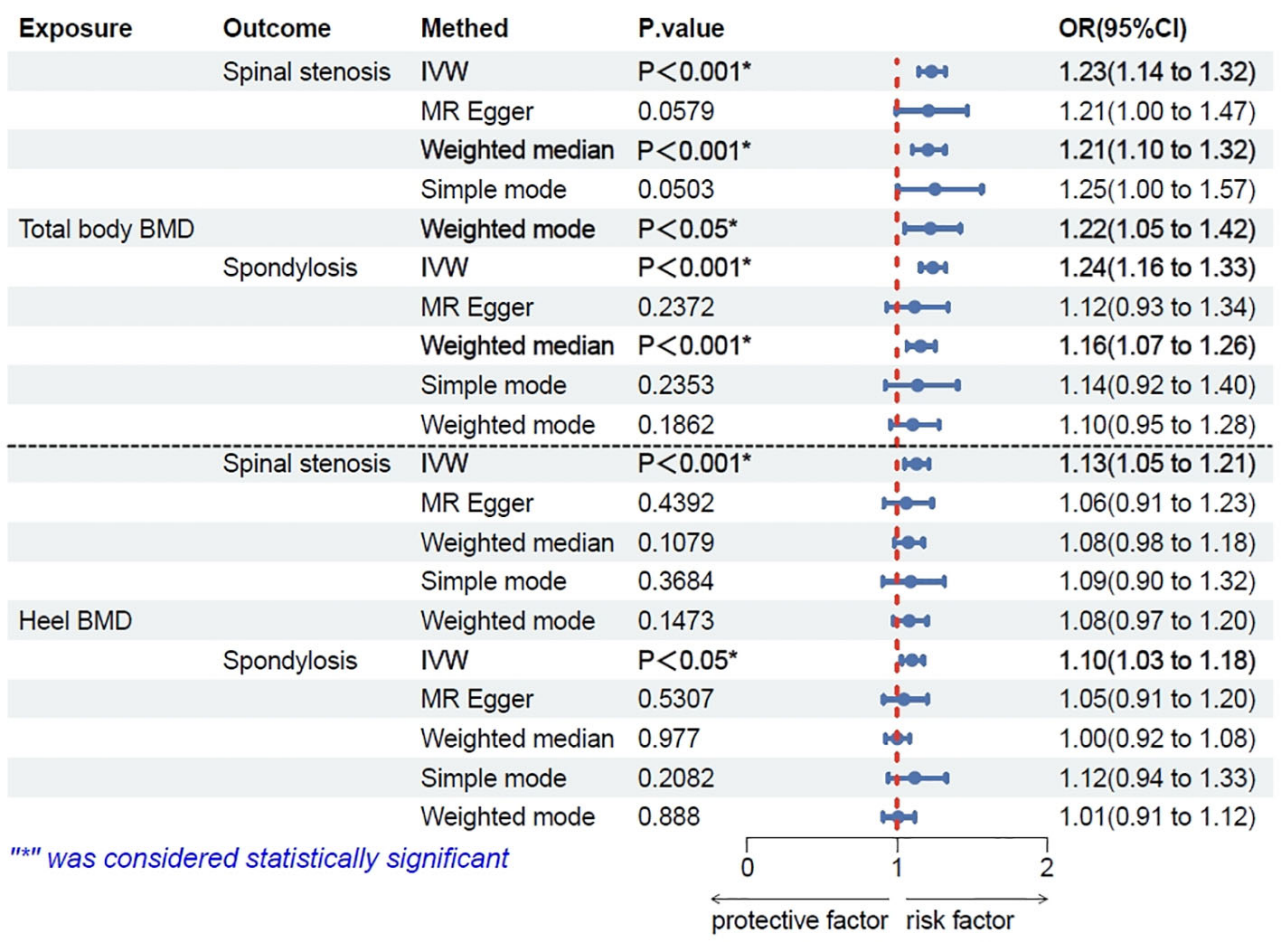
Results of Mendelian randomization (MR) analysis for total-body bone mineral density (BMD) and heel BMD single nucleotide polymorphisms (SNPs) on spinal stenosis and spondylosis though five methods.

The results showed no significant association (P > 0.05) between total-body BMD and spinal instability, spondylolisthesis, and scoliosis. However, significant associations were found with spinal stenosis and spondylosis (P < 0.001). An increase of one standard deviation (SD) in total-body BMD was associated with a 23% increased risk of spinal stenosis (IVW odds ratio [OR] 1.23; 95% confidence interval [CI]: 1.14–1.32; P < 0.001; **Figure 2**) and with a 24% increased risk of spondylosis (IVW OR 1.24; 95%CI: 1.16–1.33; P = 0.0048; **Figure 2**). In the overall IVW meta-analysis, the OR of a 1-SD increase in genetically predicted spinal stenosis was 1.13 (95%CI, 1.05–1.21; P < 0.001; **Figure 2**) and that of spondylosis was 1.10 (95%CI, 1.03–1.18; P = 0.0048; **Figure 2**).

Consistent with the above results, heel BMD was associated with spinal stenosis (P < 0.001) and spondylosis (P<0.05), but showed no significant association (P > 0.05) with other spinal disorders. Lumbar spine BMD was associated with spinal stenosis (P < 0.001) and spondylosis (P < 0.001), but showed no significant association (P > 0.05) with other spinal disorders. Compared to Total-body BMD, lumbar spine BMD yields consistent data analysis results with various spinal disorders as an exposure factor. However, these results are for reference purposes only. Please refer to the discussion section for a detailed explanation.

The results of the univariate MR analysis showed no evidence of potential weak instrument bias (all F-statistics > 10). MR-Egger analysis indicated no horizontal pleiotropy (intercept P > 0.05).

A sensitivity analysis was conducted using the leave-one-out method (**Supplementary Figure 1**), which demonstrated the robustness of the results (all points were located to the right of zero, indicating that the exclusion of any SNP did not fundamentally change the results). The funnel plot (**Supplementary Figure 1**) showed no publication or other biases (all points were evenly distributed on both sides of the effect line). The slope of the line in the scatter plot (**Supplementary Figure 1**) reflected a positive correlation of total-body BMD with spinal stenosis and with spondylosis. Each solid horizontal line in the forest plot (**Supplementary Figure 1**) represents the results estimated from various SNPs using the Wald ratio method. Taken together, these approaches demonstrated the reliability of our results.

## 4 Discussion

In this study, we used GWAS data from public databases with substantial sample sizes to investigate the association between total-body BMD and various spinal disorders, using dual-sample MR analysis. Supplementary validation tests were performed using exposure factors from other databases. In this way, we demonstrated the significant correlations of total-body BMD and heel BMD with spinal stenosis and with spondylosis, indicating that BMD is a crucial risk factor for these conditions.

Spondylosis, also known as degenerative disease, often arises from age-related changes in vertebral components, including vertebral disc calcification, facet joint instability, and ligament ossification (23). Spinal stenosis is commonly found in the cervical and lumbar regions, with the cervical region being more prevalent (24). Notably, posterior longitudinal ligament ossification is a pivotal etiological factor in this condition (25).

Numerous studies have highlighted the role of BMD in various spinal disorders, which has attracted widespread clinical attention (26–28). However, robust clinical evidence for a strong relationship between BMD and conditions of the spine remained insufficient (29). Previous studies have reported that patients with ossification of the posterior longitudinal ligament (OPLL) in the cervical spine exhibited significantly higher average BMD than did those in the control group (30, 31). Moreover, elevated levels of bone formation markers (Procollagen Type 1- Carboxy terminal propeptide [PICP] and intact osteocalcin) have been detected in the serum of patients with OPLL (32). This finding might indicate elevated overall bone formation activity in patients with OPLL, suggesting a potential link between disease progression and bone formation, which has been suggested by previous studies (33–35).

Given that the conclusions of previous studies have often been based on lumbar spine BMD data, the density measurements might be exaggerated by the ossified ligament itself (35, 36). Therefore, we do not intend to provide further explanation. A recent publication employed total-body BMD as the primary indicator of the impact of systemic bone metabolism, in a comparison between OPLL and non-OPLL patients (14, 31, 32). In agreement with our study, they found significantly higher total-body BMD in OPLL patients, particularly among middle-aged and older women and men (14). We speculate that appropriately controlling a patient’s total-body BMD at the average population level may be advantageous for managing disease progression. When an increasing trend in BMD is detected during patient examinations and if the patient complains of numbness, pain, and the sensation of walking on cotton, awareness and vigilance for the potential occurrence of conditions such as spondylosis or spinal stenosis are needed (37, 38). In particular, previous studies have indicated a significant correlation between increased BMD and lower back pain in middle-aged women (13). Follow-up spinal computed tomography scans should be conducted to assess issues, such as ligament ossification (39), and prompt treatment should be administered, as necessary (40).

The strength of this study lies in the utilization of multiple extensive GWAS summary datasets. Employing stringent quality control conditions and analytical methodologies for MR analysis, we revealed causal relationships and endeavored to minimize bias in the outcomes. Our results hold paramount significance for guiding the prevention and development of spondylosis and spinal stenosis and informing future clinical research directions. Furthermore, the robustness of our results was reaffirmed by substituting BMD data from different anatomical regions and using a larger sample size for exposure factors. Furthermore, given the paucity of research on the relationship between total-body BMD and various spinal disorders, we here used MR because of its relatively straightforward and feasible design, as compared with previous observational studies. Nonetheless, our study was limited in that the GWAS data were derived exclusively from individuals of European descent, which precludes the generalization of the results to other populations. Considering the limited research on the association between total-body bone mineral density (BMD) and spinal disorders, future investigations should delve into potential genetic-level mechanisms, expand sample sizes, and formulate clinical guidelines for the early detection and intervention of these conditions.

## 5 Conclusion

In conclusion, this study provided evidence of a positive correlation between total-body BMD and the prevalence of spondylosis and spinal stenosis, but found no association with other spinal disorders. Higher levels of total-body BMD are associated with an elevated risk of spondylosis and spinal stenosis development. Our results imply patients demonstrating an increasing trend in BMD and who have complaints of numbness and pain should be investigated for possible spondylosis or spinal stenosis and should be treated appropriately.

## Data availability statement

The original contributions presented in the study are included in the article/**Supplementary Material**. Further inquiries can be directed to the corresponding author.

## Ethics statement

All data were obtained from the IEU and the Finnish databases. The studies were conducted in accordance with the local legislation and institutional requirements. The participants provided their written informed consent to participate in this study.

## Author contributions

AS: Supervision, Writing – review and editing. QJ: Writing – original draft. HG: Formal Analysis, Writing – review and editing. XS: Methodology, Writing – review and editing. YW: Data curation, Writing – review and editing. WN: Writing – review and editing.
