# Supplementary material for: Total body bone mineral density and various spinal disorders: a Mendelian randomization study
Source: Front Endocrinol (Lausanne). 2023 Oct 31;14:1285137. doi: 10.3389/fendo.2023.1285137 (PMC10644298; doi:10.3389/fendo.2023.1285137)
Supplement: Supplementary file 1 [file DataSheet_1.docx]

Supplementary Material

Total body bone mineral density and various spinal disorders: A Mendelian randomization study

**Qingyu Jiang, Haihao Gao, Xudong Shi, Yan Wu, Wentao Ni, Aijia Shang***

*** Correspondence:** 28 Fuxing Road, Beijing, China, 100853

Email: shangaj@126.com

# Supplementary Data

## Supplementary Figures


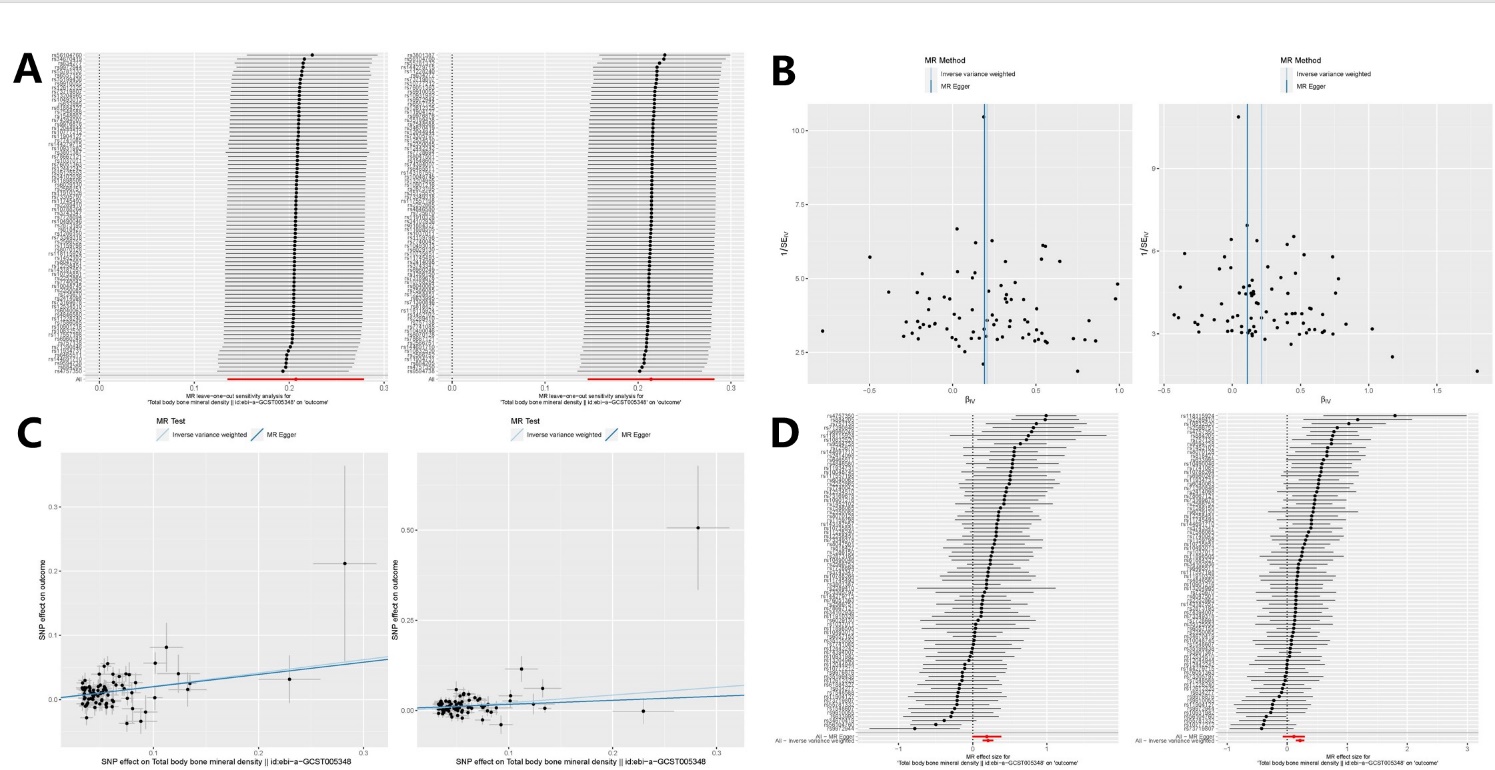


**Supplementary Figure 1.** (A) MR leave-one-out sensitivity analysis for total body BMD on spinal stenosis (left) and spondylosis (right). (B) Funnel plot of the IVW and MR-Egger models for total body BMD with potential association with spinal stenosis (left) and spondylosis (right). (C) Scatter plot of total body BMD with potential association with spinal stenosis(left) and spondylosis (right). (D) Forest plot of the causal effects of total body BMD associated SNPs on spinal stenosis (left) and spondylosis (right).
